# Childhood fussy/picky eating behaviours: a systematic review and synthesis of qualitative studies

**DOI:** 10.1186/s12966-019-0899-x

**Published:** 2020-01-03

**Authors:** Hazel Wolstenholme, Colette Kelly, Marita Hennessy, Caroline Heary

**Affiliations:** 1 School of Psychology, National University of Ireland, Galway, Ireland; 2 Health Promotion Research Centre, National University of Ireland, Galway, Ireland

**Keywords:** Fussy eating, Picky eating, Neophobia, Qualitative research, Systematic review, Meta-ethnography, Qualitative evidence synthesis

## Abstract

Fussy/picky eating behaviours are common across childhood. Recent reviews of the fussy eating literature focus on quantitative research and do not adequately account for families’ subjective experiences, perceptions and practices. This review aims to synthesise the increasing volume of qualitative work on fussy eating. A systematic search of relevant databases was carried out. Studies were included if they were qualitative, published since 2008, with a primary focus on families’ experiences, perceptions and practices regarding fussy eating, food neophobia, or food refusal in children (aged one to young adult). Studies with clinical samples, or relating to children under one year were excluded. Ten studies were eligible for this review and were synthesised using meta-ethnography (developed by Noblit and Hare). This review provides a comprehensive description and definition of fussy eating behaviours. A conceptual model of the family experience of fussy eating was developed, illustrating relationships between child characteristics (including fussy eating behaviours), parent feeding beliefs, parent feeding practices, mealtime emotions and parent awareness of food preference development. Our synthesis identified two ways in which fussy eating relates to mealtime emotions (directly and via parent feeding practices) and three distinct categories of parent beliefs that relate to fussy eating (self-efficacy, attributions and beliefs about hunger regulation). The model proposes pathways which could be explored further in future qualitative and quantitative studies, and suggests that parent beliefs, emotions, and awareness should be targeted alongside parent feeding practices to increase effectiveness of interventions. The majority of studies included in this review focus on pre-school children and all report the parent perspective. Further research is required to understand the child’s perspective, and experiences of fussy eating in later childhood.

**PROSPERO Registration:** CRD42017055943

## Introduction

Fussy/picky eating and food neophobia are common behaviours throughout childhood. Fussy eating has been defined as the consumption of an inadequate variety or quantity of foods through the rejection of a substantial amount of both familiar and unfamiliar foods [1]. Food neophobia is a related concept and refers to the unwillingness to eat new foods [1]. However, definitions of fussy eating behaviours vary widely across studies and an operational definition of fussy eating does not exist [2]. Similarly, measures of fussy eating vary considerably, resulting in inconsistent reports of the prevalence of fussy eating behaviours ranging from 5.6 to 59% [2, 3]. A recent meta-analysis of fussy eating in children over 30 months of age estimated prevalence to be 22% [4]. Although fussy eating is often reported to peak in early childhood [5, 6], the developmental trajectory of fussy eating is largely unknown [2, 7].

Despite inconsistencies in defining and measuring fussy eating, it has been associated with family stress and conflict at mealtimes as well as high levels of parent concern and frustration [4, 8]. Fussy eating and food neophobia have also been associated with child anxiety and feelings of disgust [1, 7]. Health risks associated with fussy eating are usually low [8, 9], however fussy eaters do tend to have lower intakes of vitamin E, vitamin C, folate and fibre which may lead to a weak immune response and digestive problems [1, 2].

Extensive research has been carried out on the correlates and influences of fussy eating behaviours. Child factors include age, personality, tactile defensiveness, emotionality, and cognitive factors [1, 6, 7]. Other important influences on fussy eating, food preferences and intake include genetics and environmental factors such as culture, peer influence, and media [10, 11]. Parental influence has received the most attention in the literature, particularly in relation to parent feeding practices [12], possibly due to these factors being the most amenable to intervention. Research on parent feeding practices and fussy eating has found that positive or responsive feeding (involving an awareness of hunger and satiety cues, and a division of responsibility in which parents provide the meal and the child decides how much to eat) are associated with lower levels of fussy eating, while negative or non-responsive feeding practices (such as pressure to eat and using food as a reward for behaviour) are associated with higher levels of fussy eating [4, 6, 13].

Research has suggested that raising awareness of evidence-based practices such as repeated exposure to foods would be of benefit to parents [8, 14]. However, other research suggests that knowledge alone does not always promote behaviour change [15] and that we need to explore other factors that might support parents to make changes. It has been demonstrated that there is a bi-directional relationship between parent feeding practices and fussy eating behaviours. For example, Jansen and colleagues [16] identified a bi-directional association between child fussy eating and parental pressure to eat, indicating that parents both influence and are influenced by the characteristics of their children. It has also been suggested that maladaptive practices may result from parents’ expectations and anxiety about their child eating too little, the belief that children cannot self-regulate their hunger levels, and low parental self-efficacy [4, 6, 9, 17, 18]. However, there is limited research on the role of all of these factors in contributing towards parents’ feeding practices, and the relationships between these factors and childhood fussy eating are poorly understood. A better understanding of these factors may contribute to the development of more effective interventions that target parental feeding practices.

There is increasing recognition of the importance of qualitative work in both intervention development and informing quantitative work [19–21]. Specifically, the World Health Organisation [22, 23] has highlighted qualitative evidence synthesis as a key approach to understand the needs, values, perceptions and experiences of stakeholders and to inform the development of health guidelines. In the context of fussy eating, qualitative studies provide useful insights into family mealtime experiences and parent feeding practices used to manage these behaviours. Qualitative research also highlights novel findings in relation to parents’ beliefs and motivations, which could improve our understanding of the context in which certain feeding practices are used, as well as the effectiveness of interventions aiming to resolve fussy eating related challenges.

Despite numerous reviews of the definitions, prevalence, correlates and management of fussy eating since 2008 [1–4, 6–8], these reviews focus primarily on quantitative findings and a review of the qualitative research on family perceptions, experiences and practices has not yet been carried out. Therefore, this study aims to review and synthesise the body of qualitative work carried out in this period, specifically examining family perceptions, experiences, and practices in relation to non-clinical childhood fussy eating behaviours. Specifically, our objective is to investigate the relationships between fussy eating perceptions (e.g. awareness, beliefs), experiences (e.g. manifestations of fussy eating, consequences of fussy eating, mealtime emotions), and practices (e.g. repeated exposure, pressure to eat), that have been described in recent published qualitative studies, and to develop a conceptual model representing these relationships.

## Methods

A meta-ethnographic approach (following Noblit and Hare [24] and ENTREQ [25] guidelines) was used to synthesise the qualitative literature on family experiences, perceptions and practices regarding non-clinical childhood fussy eating. Meta-ethnography is a qualitative synthesis method widely used across psychology and health care disciplines [26], and is a form of secondary analysis involving re-interpretation of published findings. Meta-ethnography aims to synthesise qualitative research while maintaining the context of each individual study, unlike a meta-analysis of quantitative literature which aims to aggregate data. A qualitative synthesis aims to establish meaning by relating knowledge from different original studies and highlighting the relevance of this knowledge to a specific topic [24]. Meta-ethnography was selected using the RETREAT (Review question; Epistemology; Time; Resources; Expertise; Audience and purpose; Type of data) framework which provides guidance on selecting a qualitative synthesis approach [19]. Specifically, meta-ethnography was well suited to our review question, quantity (and type) of data available, time frame, and target audience. Meta-ethnography following Noblit and Hare involves seven steps [24, 26], detailed below (and in Table 1).

**Table 1:** Meta-ethnography phases, steps, and tools/software used to review and synthesise studies

Phase of Review and Synthesis	Steps	Tools/Software Used
Choosing a synthesis approach	1. Select a qualitative synthesis approach appropriate for review question	RETREAT framework [19]: consider Review question, Epistemology, Timeframe, Resources, Expertise, Audience & purpose, and Type of data
Phase 1: Getting started	1. Preliminary literature searches	Databases (Embase, Scopus, PsycINFO)
	2. Register review protocol	PROSPERO (CRD42017055943)
Phase 2: Deciding what is relevant to the initial interest	1. Develop search strategy and run exhaustive search of databases	Databases searched: Cinahl Plus, Embase, Scopus, PsycINFO, Proquest (ASSIA and Sociological Abstracts)
	2. Title and abstract screening	COVIDENCE
	3. Full text screening	Microsoft Word
	4. Team discussions about discrepancies	
	5. Supplementary searches	Reference lists, author searches on Google Scholar, ‘Cited by’ tools on Scopus and Google Scholar
Phase 3: Reading the studies	1. Data extraction (full texts)	NVivo
	2. Noting initial observations	Memos in NVivo
	3. Extract key contextual information and key findings	NVivo (to organise data) Microsoft Word (to visualise data in table format)
	4. Quality appraisal	Joanna Briggs Institute Critical Appraisal Checklist [28]
Phase 4: Determining how the studies are related	1. Consider similarities and differences across studies	Matrix in NVivo Table in Microsoft Word
Phase 5: Translating the studies into one another	1. Enter key contextual information for each study to preserve context and meaning of original studies throughout the analysis process.	Microsoft Excel spreadsheet
	2. Enter metaphors (findings from each study) into table (row for each study, column for each new metaphor not already reported by a previous study) If studies reported similar findings under different names or themes, these findings were entered into the same column and a metaphor name was selected which best represented all of the data	Microsoft Excel spreadsheet
	3. Compare each study against all previous studies, observing initial similarities (reciprocal translations) and differences (refutational translations) between studies	Microsoft Excel spreadsheet
	4. Colour coding 1st order (participant quotes), 2nd order (primary study author) and 3rd order (reviewer) interpretations to preserve context and meaning	Microsoft Excel spreadsheet
Phase 6: Synthesising translations	1. Read excel file row by row summarising similarities and differences of each study (reciprocal and refutational translations)	Microsoft Excel spreadsheet
	2. Read excel file column by column to define, refine and summarise each metaphor while observing similarities and differences across studies	Microsoft Excel spreadsheet
	3. Group similar metaphors (original findings) together into 3rd order constructs (categories developed by reviewer)	Microsoft Word
	4. Develop themes that describe constructs and relationships between them	Microsoft Word
	5. Map relationships between key themes within each individual study	Conceptual models using paper and pen
	6. Integrate individual conceptual models to form an overarching conceptual model of relationships between constructs across studies	Conceptual model (Microsoft PowerPoint) (See Fig. 2)
Phase 7: Expressing the synthesis	1. Write a summary of each theme supported by quotes	Microsoft Word
	2. Illustrate findings visually	Conceptual model (Microsoft PowerPoint)
	3. Consider purpose and audience of review	
	4. Assess confidence in review findings (relationships in the model), and consider any alternative interpretations of findings	GRADE CERQual [30]
	5. Consider quality of reporting	ENTREQ [25] QMARS [20] eMERGe [31]
	6. Rewrite theme summaries considering confidence and alternative interpretations	Microsoft Word

### Phase 1: Getting started

Preliminary literature searches were carried out in 2016–17 to assess the feasibility of the review and the review protocol was registered on PROSPERO (https://www.crd.york.ac.uk/PROSPERO/ registration number: CRD42017055943).

### Phase 2: Deciding what is relevant to the initial interest

Due to the small number of qualitative studies on fussy eating, it was likely that each study would contribute new knowledge to the synthesis. Therefore, an exhaustive search of the literature (rather than a purposive search) was considered appropriate. Following preliminary database searches and two consultations with a subject librarian, a final search strategy was developed to achieve a balance between sensitivity (maximising retrieval of relevant items) and specificity (minimising retrieval of irrelevant items) [27] (search strategy details can be seen in Table 2). The search was limited to research published since 2008 because preliminary searches indicated a significant increase in research on fussy eating since 2008. In addition, the majority of qualitative studies on fussy eating had been published since 2015 and several reviews on fussy eating had been carried out in 2008 and 2015 [1, 2, 6] with limited reference to qualitative research. Extending the search beyond this time would significantly increase the number of irrelevant items to be screened with a low chance of identifying relevant articles. Given the limited number of qualitative studies on fussy eating in childhood, a broad age range was selected to maximise retrieval of relevant items that would add to our understanding of fussy eating across childhood.

**Table 2:** Search strategy used to identify qualitative studies on fussy eating in childhood published since 2008

Search Strategy	
**Search terms** (based on key words of relevant articles and test searches in Scopus and Embase. Terms and search operators varied slightly according to database guidelines)	
***Concept 1*** ***(focus)***	Fussy eat(ing/er(s)); Food W/15 (within 15 words of) fuss(iness); Picky eat(ing/er(s)); Food W/15 pickiness; Faddy eat(ing/er(s)); Finicky eat*; Choosy eat(ing/er(s)); Selective eating; Food selectivity; Neophobia; Food refusal; Food rejection; Food aversion
***Concept 2*** ***(target age)***	Child(ren); Pre(-)school(er(s)); Toddler(s); School(-)age(d); Adolescen(ce/t(s));Teen(s/age/aged/ager(s); Preteen(s/age/aged/ager); Youth(s)
***Concept 3*** ***(research method)***	Qualitative; Qualitative research; Qualitative study; Qualitative method; Interview(s/ing/ed); Focus group(s); Phone(s/call); Diary/diaries; Photo(s); Memo(s); Qualitative analysis; Thematic analysis; Content analysis; Grounded theory; Phenomenological analysis; Discourse analysis; Narrative analysis Observ(e/ed/ing/ation(s)
***Concept 4*** ***(participant)***	Parent(s/ing); Guardian(s); Caregiver(s); Mother(s); Father(s); Couple(s); Child(ren); Adolescent(s); Son(s); Daughter(s); Sibling(s); Famil(y/ies)
**Search limits**	2008-2018
**Databases** (selected to span psychology, social science and medical disciplines)	Cinahl Plus, Embase, Scopus, PsycINFO, Proquest (ASSIA and Sociological Abstracts)
**Date of final database search** (conducted by HW)	11-Jul-2018
**Supplementary search strategies**	Backchaining (searching reference lists of relevant studies), forward chaining (searching research citing relevant studies), searching other work by authors of relevant studies

**Fig. 1:**
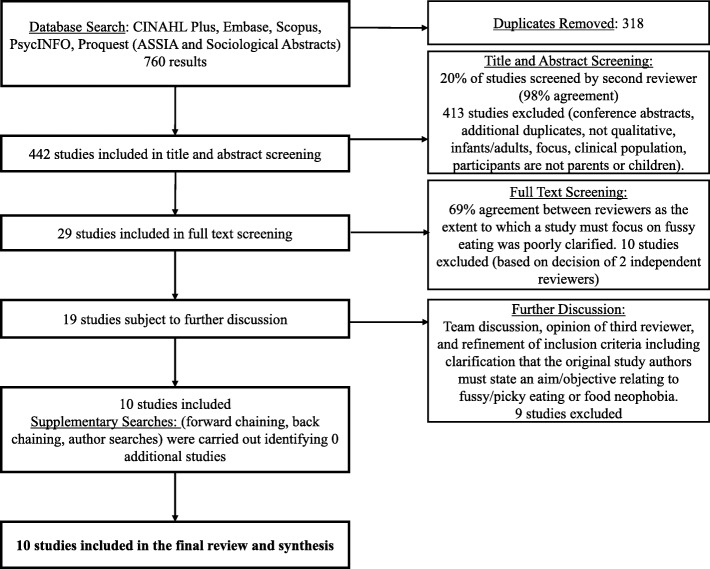
Flow chart illustrating selection of studies through database searches, screening, team discussions and supplementary searches

Title and abstract screening (HW & CH), as well as full text screening (HW & MH) were carried out based on the inclusion and exclusion criteria listed in Table 3. Supplementary searches did not identify any additional sources that had not already been identified by the database search. Additional details in relation to study selection are included in Fig. 1.

**Table 3:** Inclusion and exclusion criteria for title and abstract and full text screening

	Inclusion Criteria	Exclusion Criteria	Rationale
Methodology	Qualitative studies (using both qualitative methods and analysis) Mixed methods studies in which the qualitative component can be extracted	Quantitative studies Review articles Intervention studies (evaluations of interventions)	Mixed methods are included due to the small number of relevant studies available Qualitative evaluations of interventions are excluded in order to represent family experiences of non-clinical fussy eating prior to any intervention
Dates	Published between 2008 and July 2018	Published before 2008	Focus on recent research Searching prior to 2008 would significantly increase the number of irrelevant items to screen with a low chance of identifying relevant articles
Language	English	Any language other than English	Author resources
Target Age	Children from one year to young adult	Eating behaviours of infants less than one year and independent adults	Broad range due to limited number of studies on childhood fussy eating Wide age range would maximise retrieval of items that would contribute to our understanding of fussy across childhood Focus on children over one year as younger children are still being introduced to solid foods
Focus	Experiences, perceptions and practices regarding fussy eating/food neophobia/food rejection/refusal (min. one relevant sentence in abstract during title and abstract screening; author stated relevant aim or objective in full text screening)	Studies on: food preference without reference to fussy eating/neophobia/food refusal, breastfeeding and weaning, food insecurity, malnutrition related to poverty, intervention implementation	Diverse terminology used to report ‘fussy/picky’ eating behaviours
Context	Typically developing population	Studies on specific populations with a diagnosis of a condition impacting eating behaviour (including diabetes, cancer, autism, other disabilities, premature infants)	Studies carried out in the context of a diagnosis may not be transferable to typically developing populations
Participants	Children and parents or primary caregivers	Other family members, teachers, healthcare professionals	Focus on family experience of fussy eating behaviours

### Phase 3: Reading the studies

At this stage, full texts were imported to NVivo qualitative data analysis software, QSR International Pty Ltd. Version 11.4. The first reviewer (HW) actively read all included studies, noting any initial observations (e.g. how study findings relate to the review question, quality and quantity of data, how authors define or describe fussy eating, important contextual factors, findings of potential interest). Key contextual information was extracted from the introduction and methods sections of each paper (Table 4). First order (participant quotes) and second order (author) interpretations were extracted from results and discussion sections.

The first author (HW) used the Joanna Briggs Institute Critical Appraisal Checklist for Qualitative Research ([28], https://joannabriggs.org/critical_appraisal_tools) to assess the quality of each individual study (reported in Table 4). This was to aid interpretation of findings at later stages of the review and studies were not excluded on the basis of poor quality. Studies were of moderate-high quality (average 7.15/10). Most studies failed to provide information regarding the philosophical perspective of the study and the impact of the researcher on the research. However, this is likely due to space limitations in publications and may be representative of the published report rather than the quality of the research [26].

### Phase 4: Determining how the studies are related

At this stage, key findings and study characteristics were presented in table format in MS Word (similar to Table 4). The first reviewer (HW) considered similarities and differences across studies in relation to contextual factors such as country, sample, and socio-economic status of participants. These observations increased the reviewer’s familiarity with the contexts of each study prior to analysis, and determined the order in which studies would be analysed in Phase 5 (detailed below in Phase 5).

### Phase 5: Translating the studies into one another

The process of translation aims to maintain the central findings of each study (referred to as metaphors), while also comparing the findings in one study with those in the other studies [24]. The key steps involved in this phase are detailed in Table 1. The process started with findings from Rubio and colleagues [29] as this study was considered to have the highest quality and quantity of relevant data based on initial observations in Phases 3 and 4. Studies were then entered one by one into the Microsoft Excel file (by HW) according to study characteristics, to maximise proximity of studies with similar contexts (e.g. studies using the same sample, low-income samples, school-aged children, see Table 4). Translating studies into one another was an iterative process in which previous studies were continuously re-read to look for any data to support newly identified metaphors which may have been overlooked during previous readings. The process of extracting metaphors from studies, and the final excel file were reviewed by a second reviewer (CH) and discussed by the review team.

### Phase 6: Synthesising translations (developing a line-of-argument synthesis)

**Fig. 2:**
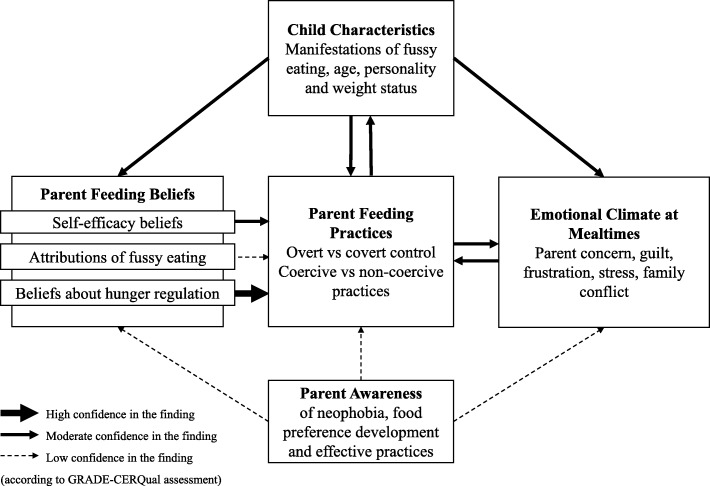
Conceptual model illustrating 5 constructs (and how they relate to one another) generated by a secondary analysis of findings in recent qualitative studies

Similar to primary qualitative research moving from descriptive to explanatory, this phase involves moving from translations (produced in Phase 5) to a higher order interpretation, or a ‘line-of-argument’ [26] and creating a whole picture which represents more than the individual parts alone imply [24]. Steps involved in this phase are detailed in Table 1. Third-order constructs (categories of findings/metaphors generated by the reviewer), themes (text explaining constructs and how they relate) and the conceptual model (Fig. 2) were derived inductively. The process was reviewed by members of the review team (CH, CK), who offered alternative views and interpretations. Themes and conceptual maps were refined following team discussions (HW, CH, CK).

### Phase 7: Expressing the synthesis

The line-of-argument synthesis was expressed by writing a summary of each theme (outlining the five constructs and relationships between them), supported by both first order (participant) and second order (author) quotes from primary studies, and by developing a conceptual model which illustrates the relationships between constructs. The purpose of this review is to contribute to our theoretical understanding of fussy eating behaviours, therefore it is targeted at an academic audience. It is expected that findings will also be applicable to policy makers, practitioners and intervention developers.

GRADE-CERQual (Confidence in the Evidence from Reviews of Qualitative Research) [30] was used to assess the extent to which the findings from our synthesis (i.e. relationships between constructs illustrated in our model) are a reasonable representation of the phenomenon of interest based on methodological limitations, coherence, adequacy and relevance of the data supporting each finding. Confidence in each finding is indicated in Fig. 2 and additional information is provided in Table 5. Qualitative Meta-Analysis Reporting Standards (QMARS) [20] guidelines were consulted to ensure American Psychological Association (APA) guidelines for reporting qualitative meta-analytic research were met. Specific guidelines for reporting meta-ethnography (eMERGe [31]) and for enhancing transparency in reporting the process of synthesising qualitative research (ENTREQ [25]) were followed. We also ensured that our report was representative of the original research articles by grounding findings in the texts by providing supporting quotes and referring to the contexts of original studies throughout [32].

## Results

### Study characteristics

As can be seen in Fig. 1, ten studies were included in the final review. The characteristics of these studies are presented in Table 4. Studies represented a total of 372 parents or primary caregivers from 8 datasets (studies E and F used the same dataset, and studies I and J used the same dataset). One study (C) represented mothers only. All other studies included both female and male caregivers, however only 29 fathers took part (approx. 8% of the total number of participants). Seven studies (from 6 datasets; A, B, C, D, E/F, G) focused on preschool children aged between 1 and 5 years. Three studies (from 2 datasets; H, I/J) focused on a broader age-range including parents of children aged 1.5–21 years. Half of the studies focused on low-income families or geographical areas of deprivation (A, B, C, I/J), and half represented diverse socio-economic backgrounds (D, E/F, G, H). Two of the included studies (C & H) were mixed-method studies, and in these cases only qualitative findings were included. Study D included both infants (under one year) and toddlers (over one year), however, only sections of the paper relating to the toddler group were included in this review.

**Table 4:** Characteristics of original qualitative studies synthesised in this review

Study	Country and Author Disciplines	Age Group Targeted	Sample/Population	Aims/Objectives	Data Collection, Analysis, & Summary of Interview Guide	Quality Appraisal^a^	Key Findings Reported by Primary Study Authors
Study A Rubio et al. 2017 [29]	France Psychology	Pre-schoolers 18–38 months	38 parents (35 mothers, 3 fathers) General community sample Low-moderate income Recruited through day care centres	To explore parental concerns about their toddler’s pickiness and its consequences for parent-child relationship and family meals. To understand parental attributions of food pickiness and to investigate how parents manage their children’s food refusals.	Focus groups Thematic analysis Interview guide: Onset of child’s eating difficulties, parental perceptions and beliefs, parental strategies and food practices.	Moderate	The majority of parents report changes in food behaviours. Parents feel responsible. Picky eating causes parental anxiety and guilt. Attributions include opposition. Variety of different practices including repeated exposure, modelling and rewards for eating.
Study B Goodell et al., 2017 [55]	US Nutrition Sciences; Pediatrics; Human Development	Pre-schoolers 3–5 years	111 primary caregivers (104 female, 6 male, 1 chose not to answer) Low-income African American and Hispanic parents Recruited from Head Start Centers	To determine parent feeding strategies used to influence child acceptance of previously rejected foods.	Focus groups Thematic analysis Interview guide: Several topics relating to child feeding and mealtimes including: what strategies do parents use to influence their children to like previously rejected foods?	High	Parents often do not serve previously rejected foods. Parents value their child eating over liking a food. Parents rarely use the same feeding strategy more than once for a previously rejected food. Parents wish to reduce waste, save time, and ensure children eat enough for adequate growth.
Study C Jarman et al., 2015 [56]	UK Lifecourse Epidemiology; Nutrition Biomedical Research; Psychology; Musculoskeletal Biomedical Research	Pre-schoolers 18 months – 5 years	29 mothers Socially deprived area Purposive sampling	To explore mothers’ use of overt and covert control practices (and relationship with neophobia). Specifically, what do mothers say about controlling their children’s eating habits?	Mixed method Focus groups Thematic analysis Interview guide: Not provided	High	Feeding young children is stressful. Parent control is often relinquished to reduce conflict at mealtimes.
Study D Harris et al., 2018 [50]	Australia Children’s Health; Exercise & Nutrition Science; Social Science	Pre-schoolers 1–4 years	6 parents of children > 1 year (5 female, 1 male) General sample, mix of low and high socio-economic status	To characterise parents’ presentation of fussy eating and mealtime interactions at a point of crisis.	Calls to a help-line Inductive thematic analysis Interview guide: n/a	Moderate	Parents of toddlers present emotional accounts of feeding, portrayed their child’s eating behaviours as a battle and child agency over intake/variety as ‘bad’ or ‘wrong’. Escalating concern evoked non-responsive feeding practices.
Study E Russell et al., 2013 [33]	Australia Exercise & Nutrition Sciences	Pre-schoolers 2–5 years	57 parents (49 female, 8 male) General community sample recruited from a range of SES background Purposefully selected from survey participants	To describe parents’ beliefs (attributions and self-efficacy) about the origins of children’s food preferences that may influence parental feeding behaviours. To examine differences between parents of children with healthy preferences, unhealthy preferences and neophobia.	Interview Content analysis Interview guide: describe child’s likes and dislikes, influences of preferences, how much preferences change over time, how much influence parents have over child preferences.	Moderate	Attributions of food preferences include child characteristics, sensory attributions, and socialisation experiences. Beliefs (and self-efficacy) differ between parents of children with healthy preferences, unhealthy preferences, and neophobia supporting the idea of causal links between parent beliefs, behaviours, and child characteristics.
Study F Russell et al., 2015 [57]	Australia Health; Exercise & Nutrition Sciences	Pre-schoolers 2–5 years	57 parents (49 female, 8 male) General community sample recruited from a range of SES background Purposefully selected from survey participants	To describe behaviours used by parents to influence children’s food preferences. To examine differences between parents of children with healthy preferences, unhealthy preferences and neophobia.	Interview Content analysis Interview guide: behaviours used to influence children’s preferences (likes and dislikes), whether methods were effective and why.	Moderate	Parents used diverse behaviours to influence their child’s food preferences. Parents of children with healthy preferences appeared to use more effective feeding behaviours. Parents of children with unhealthy and neophobic preferences appeared to use more ineffective behaviours.
Study G Norton et al., 2016 [58]	Australia Business	Pre-schoolers 1–2.5 years	24 parents (23 female, 1 male) General community sample recruited from range of socio-economic areas Snowball sampling and purposeful selection	To explore primary caregivers’ awareness of food neophobia and how food preferences develop in young children.	Interview and projective technique drawings Cross case analysis Interview guide: history of child’s eating, foods that should be provided to a child on an everyday basis, other foods. Drawings of crying child in a trolley and child making a mess in a highchair.	Moderate	Primary caregivers are unaware of food neophobia and food preference development in young children.
Study H Boquin et al., 2014 [35]	US Food Science & Human Nutrition; Market Research	Children 18 months – 21 years	19 parents (14 female, 5 male) General sample	To investigate perceptions of picky eating. To determine the most predictive elements that people use to describe a picky eater.	Mixed method Focus groups Analysis method described but not specified Interview guide: describe mealtimes, picky eating perceptions, definitions and characterisations.	Moderate	Fussy eaters display before mealtime behaviours (being uninterested or avoidant), during mealtime behaviours (being disengaged, uninvolved, distracted, carefully inspecting food, having strong physical reactions to foods), general mealtime preferences, and food sensory-dependent preferences. Top two perceptions of picky eating: 1) unwilling to try new things, 2) consuming limited type and amount of food.
Study I Trofholz et al., 2017 [34]	US Family Medicine & Community Health	Children 2–18 years	88 parents (83 female, 5 male) Racially and ethnically diverse Low-income sample Recruited from previous study	How do parents describe child picky eating? How do parents perceive picky eating to impact the family meal? How do parents report responding to picky eating in the family meal?	Interview Content analysis Interview guide: what kind of eater child is, how eating impacts meal, how picky eating affects the family, what happens if child doesn’t want to eat what is prepared, how parents influence what child eats.	High	Children are frequently described as picky eaters, parents define picky eating in a variety of ways, picky eating impacts the family meal (stress, meal preparation), parents respond in a variety of ways.
Study J Berge et al., 2016 [36]	US Family Medicine & Community Health; Human Development & Family Studies; Epidemiology & Community Health	Target children 6–12 years Siblings 2–18 years	88 parents (83 female, 5 male) Racially and ethnically diverse Low-income sample Recruited from previous study	How do parents describe their approach to feeding siblings? Do parents engage in different feeding practices based on child-specific characteristics (weight, picky eating, age, sex, temperament)?	Interview Content analysis Interview guide: what it is like to be a parent of two (or more), how you decide what to feed your children, how do you feed them (similarly and differently), role as a parent during mealtimes, how you influence what siblings eat (child characteristics)?	High	Food preferences, in-the-moment decisions and planned meals influence decisions about what to feed siblings. Picky eating is managed by making one meal or by giving leeway to siblings about having other food options. Parents used different feeding practices.

^**a**^JBI Critical Appraisal Checklist. Assessment is based on 10 items regarding congruity between authors’ philosophical perspective, methodology, methods, research question and data analysis, the interpretation of results, the influence of the researcher on the research, adequate representation of participant’s voices, ethics, and conclusions drawn from the analysis. Moderate indicates a score of 5–7. High indicates a score of 8–10

### Line-of-argument synthesis (building a whole picture from the individual parts)

Translating the ten studies into one another (Phase 5 of the analysis) produced 54 metaphors (individual findings identified by the primary study authors). In Phase 6 of the analysis, the first reviewer (HW) grouped similar metaphors together to produce 21 third-order constructs (sub-categories identified by the reviewer). These third-order constructs were categorised further (by HW), to produce five main constructs (child characteristics, parent feeding beliefs, parent feeding practices, emotional climate at mealtimes and parent awareness of neophobia, food preference development and effective practices). Five themes were developed that explain these constructs and how they relate to one another. Together, the final five constructs and five themes form an overall line-of-argument synthesis represented by the conceptual model in Fig. 2.

Overall, this model describes and explains the family experience of fussy eating behaviours (as indicated by the current qualitative literature), and proposes relationships between childhood fussy eating behaviours, parent feeding beliefs, parent feeding practices, mealtime emotions and parent awareness of food preference development. As highlighted in the model, there is higher confidence in some relationships over others, indicating better quality (and quantity of) data supporting these findings, as assessed using the GRADE-CERQual assessment tool [30]. The GRADE-CERQual assessment for each finding is detailed in Table 5.

**Table 5:** GRADE-CERQual assessment: confidence that relationships in the model are a reasonable representation of the phenomenon of interest

Summary of Review Finding (Relationship in Fig. 2)	Studies Contributing to Review Finding	Methodological Limitations^a^	Coherence^b^	Adequacy^c^	Relevance^d^	CERQual Assessment^e^	Explanation of CERQual Assessment
Theme 1 & 2: Child characteristics (including fussy eating behaviours) and parent feeding practices							
Parent feeding practices have an impact on child fussy eating behaviours (either by overcoming, or reinforcing behaviours).	A, B, C, D, E, F, G, I, J	No or very minor concerns that all coded parentfeeding practices were adequately reported in Study B. However finding is reported across studies with diverse methods.	Minor concerns due to some cases where parents do not effectively influence the child and the possible influence of other factors such as knowledge and self-efficacy (D, E, G).	Minor concerns that although impact of parents on child behaviours are often assumed by authors, there was a lack of quotes illustrating direct effectiveness of parent feeding practices on child fussy eating behaviours (A, C, D, G, I).	Minor concerns that this finding is specific to mothers. Some studies have a broader focus (e.g. on food preferences rather than fussy eating specifically). This finding was represented across diverse countries, contexts, income levels, ethnicities, and age-groups.	**Moderate confidence**: It is likely that the review finding is a reasonable representation of the phenomenon.	There are some minor concerns regarding some disconfirming cases, potential influence of other factors, the lack of examples illustrating the effectiveness of practices, and that this finding is specific to mothers. However this finding was reported across diverse contexts.
Child characteristics (including pickiness, weight and temperament) impact parents’ use of feeding practices.	A, B, C, E, F, G, H, I, J	No or very minor concerns. This finding was reported across many studies with different data collection and analysis methods.	Minor concerns that in some cases the relationship may be explained by other factors (such as concern and conflict) (A, B) and some disconfirming cases where parents do not feed siblings differently (J).	Minor concerns regarding lack of specific examples/quotes in some studies (A, C, E, G, H).	No or very minor concerns that this finding is specific to mothers. This finding was identified across diverse countries, contexts, income levels, ethnicities, and age-groups.	**Moderate confidence**: It is likely that the review finding is a reasonable representation of the phenomenon.	Despite minor concerns regarding some disconfirming cases, the potential influence of other factors, and lack of examples/quotes in some studies this finding was identified across many studies with diverse methods and contexts.
Theme 3: Fussy eating behaviours, parent feeding practices and emotional climate at mealtimes							
Manifestations of fussy eating (such as limited variety or quantity of food, and gestures such as pushing the plate away) are directly related to negative parent emotions such as frustration and concern.	A, C, D, H, I	Minor concerns that focus groups in studies A, C, H may impact parents’ discussions regarding emotions and parents may provide more emotional accounts when calling a helpline (D).	Minor concerns that fussy eating may not always contribute to negative emotions and may depend on other factors such as parent feeding practices and severity of fussy eating (H).	Minor concerns regarding lack of quotes supporting this finding (C, H) and lack of explanation of disconfirming cases in which mealtime emotions were not impacted by fussy eating behaviours (I).	No or very minor concerns. Finding may be specific to mothers. Studies focus on impact of fussy/picky eating and represent diverse countries, contexts, income levels, ethnicities, and age-groups.	**Moderate confidence:** It is likely that the review finding is a reasonable representation of the phenomenon.	Although there are some minor concerns regarding the impact of data collection methods on discussions of emotions, the potential influence of other factors, and a lack of supporting quotes in some studies, this finding was reported across diverse contexts.
Parent feeding practices relate to the emotional climate at mealtimes (for example pressure to eat may be associated with conflict).	A, F, H, I, J	No or very minor concerns regarding use of focus groups (A, H) which may impact discussions about emotions.	Minor concerns that this is an over simplified finding and the direction of influence is not clear in some examples (H), and there are some disconfirming cases (I).	Minor concerns regarding richness of data contributing to this finding in some studies (A, H).	Minor concerns that this finding is specific to mothers. This finding was identified across diverse countries, contexts, income levels, ethnicities and age-groups.	**Moderate confidence:** It is likely that the review finding is a reasonable representation of the phenomenon.	Despite minor concerns that this is an over-simplified finding, and thin data in two contributing studies this finding was identified across diverse contexts.
Emotional climate at mealtimes (such as concern, anxiety, conflict and stress) impacts parents’ choice of feeding practices (e.g. cooking alternative meals).	B, C, D, F, H, I, J	No or very minor concerns (regarding influence of focus groups and calls to helpline on reporting emotions). However the finding was reported across studies with diverse methods.	Minor concerns due to some disconfirming cases where parents are persistent in their practices and not influenced by conflict/emotions) (I, J).	Minor concerns regarding lack of specific examples/quotes and reliance on author interpretations in some studies (D, H).	Minor concerns that this finding may be specific to mothers however this finding was identified across diverse countries, contexts, income levels, ethnicities and age-groups.	**Moderate confidence:** It is likely that the review finding is a reasonable representation of the phenomenon.	Although there were some concerns regarding some disconfirming cases and lack of specific examples/quotes this finding was identified across diverse contexts.
Theme 4: Fussy eating behaviours, parent feeding beliefs and parent feeding practices							
Manifestations of fussy eating relate to parent feeding beliefs (for example, if a child refuses mushy food, fussy eating may be attributed to sensory sensitivity, or if a parent is faced with a highly neophobic child, they may experience lower self-efficacy).	A, D, E, H, I	No or very minor concerns.	Minor concerns that the direction of the relationship is not always clear and is not explicitly stated in some studies.	Minor concerns regarding reliance on second and third order interpretations in some studies.	No or very minor concerns that this finding is specific to mothers. These studies are relevant to this finding focusing on descriptions and attributions of fussy eating.	**Moderate confidence:** It is likely that the review finding is a reasonable representation of the phenomenon of interest.	There are minor concerns as this relationship is not explicitly stated in some studies and there is a reliance on second and third order interpretations.
Parent self-efficacy relates to parent feeding practices.	B, C, E, F, I, J	No or very minor concerns that focus groups in studies B & C may impact discussions on self-efficacy.	Minor concerns that the relationship could be explained by other factors (e.g. child’s response to foods).	Minor concerns regarding adequacy of specific quotes illustrating this finding, and reliance on second order (author) and third order (reviewer) interpretations from studies E and F.	Minor concerns that this finding is specific to mothers, and that only one study specifically focuses on self-efficacy.	**Moderate confidence:** It is likely that the review finding is a reasonable representation of the phenomenon of interest.	There are minor concerns that this finding is over simplified and also influenced by other factors. Only one study specifically focused on self-efficacy (E) so there is a reliance on second and third order interpretations.
Attributions (perceived influences) of fussy eating relates to parent feeding practices.	A, E, F	No or very minor concerns.	Minor concerns that this is an oversimplified finding as there as some disconfirming cases (E).	Moderate concerns regarding reliance on second and third order interpretations with limited quotes clearly illustrating a link. There are a small number of studies contributing to this finding.	Minor concerns that this finding is specific to mothers of pre-schoolers.	**Low confidence:** It is possible that this review finding is a reasonable representation of the phenomenon of interest.	There were some disconfirming cases, and a reliance on second and third order interpretations as well as a limited number of studies contributing to this finding.
Beliefs about hunger regulation relate to parent feeding practices. For example, parents who believe it is the parents’ responsibility to ensure their child eats (“you can’t let them starve”) may cook alternative meals. However, parents who believe it is the child’s responsibility to regulate their hunger levels (“they will eat when they are hungry”) are more likely to just cook a meal.	A, B, D, G, I, J	No or very minor concerns.	No or very minor concerns that different definitions of fussy eating in study I may impact the extent to which parents have to adapt a meal in order for their child to eat.	Minor concerns regarding reliance on author interpretations (D) and the lack of specific examples/quotes in some studies (A, G).	No or very minor concerns that this finding is specific to mothers.	**High confidence:** It is highly likely that the review finding is a reasonable representation of the phenomenon.	Although there were minor concerns regarding the data adequacy in some studies, and that this finding may be specific to mothers, this finding was reported across studies representing different countries, age groups, ethnicities, and income levels.
Theme 5: Parent awareness of food preference development and effective feeding practices: Possible associations with beliefs, practices and emotions							
Parents’ lack of awareness of neophobia, food preference development and effective practices relates to their feeding beliefs, practices, and emotions.	A, G	Minor concerns that purposeful sampling (G) and narrow age range (1–2.5 years) may result in the selection of parents who are less aware of neophobia, food preference development and effective practices.	Moderate concerns regarding potential for other factors to explain the relationship and insufficient data to fully explain this finding.	Moderate concerns regarding richness and quantity of data to support this finding, as well as a limited number of studies contributing to this finding.	Minor concerns that this finding may be specific to parents of young children, and only A study (G) specifically focused on parent awareness.	**Low confidence:** It is possible that this review finding is a reasonable representation of the phenomenon of interest.	There were moderate concerns regarding coherence and data adequacy. In addition this finding was only identified in studies with parents of young children so may not be generalizable to all parents.

^a^**Methodological limitations**: Concerns about the design or conduct of primary studies that contribute evidence to an individual review finding; ^b^**Coherence**: how clear and cogent the fit is between the data and a review finding; ^c^**Adequacy**: The degree of richness and quantity of data supporting a review finding; ^d^**Relevance**: Extent to which the body of evidence is applicable to the context specified in the review question; ^e^**CERQual assessment** categories: high confidence, moderate confidence, low confidence, very low confidence (Lewin et al., 2018)

The five themes below provide an in-depth explanation of each of the constructs and relationships identified in Fig. 2. Studies are referred to as Studies A-J in the order that they were translated into one another (Phase 5 of the analysis), and in the order presented in Table 4. Quotes in regular font represent second order (author) interpretations, and quotes in italics represent first order (participant) interpretations.

### Theme 1: Manifestations of fussy eating behaviours

Studies indicated that a significant group of parents experience changes in their toddlers’ eating behaviours such as food refusal and pickiness (Studies A, G, H, I). Study C, which focused on eating habits and control practices of mothers in an area of social deprivation, reported that “fussy eating or neophobic tendencies seemed to be the main [feeding] issues” (C). Fussy eating behaviours often began in toddler years (Studies A, E, G, H) and appeared suddenly with no explanation (A, E) “*He used to eat everything and overnight he started to be difficult*” (A). Although some parents of younger children expected fussy eating to improve with age (E), our synthesis found that across studies with parents of older children (H, I, J) fussy eating behaviours were still common, and parents of older picky eaters said that “*their children’s food preferences/avoidances lasted as the children got older*” (H).

Although some studies highlighted the impact of fussy eating behaviours on certain food groups such as vegetables and meats (A, H, I), data across studies demonstrated that all food groups could be affected, including foods such as vegetables, fruit, dairy, meat, eggs, sauces, pizza and burgers (Studies A B, E, H, I). In particular, issues were reported in relation to new foods (A, E, H, I), “*she’s kind of picky when it comes to trying things new*” (I). Parents referred to both a limited intake of foods (D, H, I, J) “*I don’t think she eats enough*” (J) and a limited variety (A, D, H, I), “*he won’t eat fruit, he won’t eat vegetables, he won’t eat potatoes, he won’t eat meat*” (D). Fussiness also manifested as frequent changes in preferences (E, I), requiring particular preparation or presentation (“*he’ll have the noodles in a separate bowl“* (E)) (E, H, I), general disinterest and avoidance of food (H), and variability in behaviours depending on context (e.g. *“the meal goes better with his grandparents”* (A)) (A, E). Study A reported specific fussy eating behaviours or reactions to foods displayed by pre-schoolers, which were supported by participant quotes in other studies of both younger and older children. These behaviours included inspecting and picking out foods on the plate (A, H, I), expressing dislikes through verbal reactions (A, B, E, F, G, I, J), gestural reactions such as pushing the plate away (A, C) and mouth-based reactions such as spitting or gagging (A, H).

### Theme 2: Child characteristics and parent feeding practices

As can be seen in Fig. 2, there was moderate confidence in a relationship between child characteristics (including fussy eating behaviour) and parent feeding practices, and both parents and children changed their behaviours in response to each other.

In response to the child’s fussy eating behaviours described in Theme 1 above, parents used a wide range of parent feeding practices in an attempt to influence their child’s eating behaviour. These included practices such as covertly influencing food availability and role modelling as well as more coercive practices such as pressure to eat and using rewards or punishments (Studies A, B, C, D, E, F, G, H, I, J). Parents tried different strategies across and during meals (B) with differing levels of success (B, C, F, H, I, J). Parent feeding practices were widely discussed across studies, with the assumption that they influence children’s eating behaviours. For example authors referred to parent feeding practices as strategies “to overcome their children’s food refusal” (B) or “to influence their children’s food preferences” (F). However, our secondary analysis of the data presented in these studies revealed a limited number of specific examples (or quotes) illustrating changes in child eating behaviours as a result of parent feeding practices.

Instances of parent feeding practices being successful in overcoming food refusal were observed in six studies (A, B, C, F, I, J) for example “*I make her taste everything…I had her taste some chicken today, ‘It looks nasty!’ But she loved it*” (I) and “*she was like “oh, what’s this green stuff” but now she eats it quite happily. So that’s taken about four weeks to wean her into that*” (F) and “*I cook with her, it works incredibly well*” (A). On the other hand, three studies provided specific examples of parent feeding practices reinforcing and maintaining the child’s fussy eating behaviours (D, E, F). For example, Study E reported that children disliked some foods “because they had been offered an alternative to eating them when they had originally expressed a dislike” and that parents believed that indulging children’s desires increased their dislike of rejected foods.

There were more specific examples and quotes illustrating changes in parent feeding practices due to their child’s fussy eating behaviours and other characteristics including individual tastes, weight, personality and age (A, B, C, E, F, G, H, I, J). This was particularly evident in Study J which focused on how parents feed siblings similarly or differently. Approximately half of the parents in this study reported feeding siblings differently (e.g. by using pressure) depending on their individual characteristics (such as weight), for example ““*you need to eat it, eat it all.” Because I don’t think she eats enough…she is too skinny compared to her sister*” and “*I feed them [siblings] different because they have different personalities and food preferences*” (J). This was also evident in Study E when a parent’s response was influenced by the child’s personality (“*There’s no point fighting with him ‘cos he’s as stubborn as they come”*)*.* Other studies emphasised that a parent’s use of feeding practices (e.g. repeated exposure or offering alternatives) was affected by whether their child had accepted or rejected the food in the past, and the “parent’s ability to cope with their child’s reactions to foods” (F). For example “*I’ve got children that attack each other, are disrespectful and trash the home. So really one more fight about food, I’m not up for it*” (C). Parents’ ability to cope with children’s food refusals was also impacted by time constraints and concern about food waste (B, F, I, J), which was particularly evident in low-income samples. For instance mothers in Study B reported not offering previously rejected foods because *“Ma don’t have time for this…I can’t afford for you [child] to go to bed hungry”* and “*I don’t want to waste it*” (B).

### Theme 3: Fussy eating behaviours, parent feeding practices and emotional climate at mealtimes

Our synthesis identified two ways in which fussy eating contributes to negative mealtime emotions. It was found that fussy eating can directly relate to parents’ negative emotions and can also contribute to negative mealtime emotions via parent feeding practices.

There was moderate confidence based on a GRADE-CERqual assessment (See Table 5), that these fussy eating behaviours can have a direct impact on parent emotions such as concern, frustration and guilt (A, C, D, H, I). For example, one study reported that “parents were afraid that the lack of food diversity might prevent their child growing” (A). High levels of concern were evident across many studies (A, B, D, H plus additional quotes in E, F, I, J). Specific fussy eating behaviours (described in Theme 1) were also associated with negative emotions. For example, one parent said “*I find it very hard…she will push her plate away and she will have a real tantrum and she won’t eat. And that, really, well, it does get to me*” (C).

In addition to a direct relationship between fussy eating behaviours and parents’ emotions, the GRADE-CERQual assessment indicated moderate confidence that parent feeding practices (described in Theme 2) relate to the mealtime emotional climate, and that parents adjust their practices to reduce stress and conflict.

For example, pressuring or forcing a child to eat was associated with a negative mealtime environment and tricky parent-child relationships (Studies A, F) “*we have screaming matches sitting at the table for three or four hours*” (F). Catering to children’s requests and cooking alternative meals was also considered stressful (F, I, J) “*It can be stressful, especially if I had a busy day…it can be kind of stressful when I have to cook something totally different…because she’s very picky*” (I). In contrast to this, some parents reported accommodating children’s preferences to be rewarding and to result in less conflict (H) and positive emotions “*It’s work, but it’s a lot of fun work, you know, and I just like to see them happy eating. It does my heart good, yeah*” (J). Although 56 out of 88 participants in Study I reported having a fussy eater, only 36 parents found it was disruptive to family meals, indicating a significant group exists who do not find fussy eating disruptive. There was insufficient data to explain why it was not disruptive in many cases, but authors suggested this may be due to parent feeding practices and the way parents have adapted to fussy eating behaviours, or this may reflect less severe fussy eating behaviours (e.g. refusal of a few foods) that are not perceived to be frustrating by parents (J).

This idea that parents adapt their practices to avoid conflict and stress at mealtimes was supported by many studies (B, C, D, F, H, I, J). Parents changed their practices to reduce stress levels. For example one parent said “*I used to make different meals for the kids but it took too much time and was really stressful for me. No, we don’t do that, not anymore*” (J). High levels of conflict often resulted in parents relinquishing control and catering to child requests (B, C, I) for example “*if the kid straight up won’t eat and she’s been screaming and yelling at you for an hour, we give in*” (I). In addition, practices were also associated with other emotions such as concern and anxiety, for example authors of Study D stated that “escalating parent anxiety (parent concern) had evoked parent non-responsive feeding practices or provision of foods the child preferred”.

### Theme 4: Fussy eating behaviours, parent feeding beliefs and parent feeding practices

By synthesising findings across studies, we identified three key beliefs that relate to parent feeding practices in the context of fussy eating: self-efficacy beliefs, attributions of fussy eating, and beliefs about hunger regulation. These parent beliefs likely develop in response to a child’s eating behaviours (Studies A, D, E, H, I), for example if a parent is faced with a highly fussy eater, they may experience low self-efficacy, may attribute fussy eating to child characteristics like sensory sensitivity and may start to believe that their child cannot regulate their own hunger.

#### Self-efficacy beliefs

Parents’ beliefs about their ability to influence their children’s eating behaviours varied. In several studies, authors indicated that some parents experienced low self-efficacy or feelings that they were doing something wrong (Study A, D, E). Low self-efficacy was also evident in some participant quotes in studies B, I, and J, for example one parent said “*I just didn’t bother to give [it] to him…I knew he wasn’t going to eat [it]”* (B). In contrast, some parents expressed higher self-efficacy, reporting higher feelings of control over their child’s behaviours (E, F). In one study, authors indicated that parents felt more able to control food intake than preferences (E), and more able to get children to like foods, than to dislike foods *“you can overcome dislikes. But with likes, there are some things they’re going to like regardless”* (E/F). Parents “internalised the child’s food intake as a reflection of their own parenting” (A, D), indicating that the way fussy eating manifests (e.g. limited variety or quantity described in Theme 1) may impact parents’ self-efficacy beliefs.

#### Attributions of fussy eating

Parents frequently attributed fussy eating to sensory sensitivity or sensory characteristics of food such as taste, texture, appearance and smell (A, D, E, H, I); “*she doesn’t like strong flavours* (A); “*he really don’t like mushy food*” (I). Fussy eating behaviours were also attributed to non-modifiable factors such as child temperament, personality (A, E), and innate or universal preferences (E, F) (“*It’s her nature*” (A) “*Generally speaking children start off liking a lot of plain foods and probably sweet foods*”(E). In addition, fussy eating was attributed to modifiable environmental factors such as parent socialisation, peer and TV influence (E, H), depicting the belief that “*you can educate your taste buds*” (E). Again, these attributions likely relate to how a child’s fussiness manifests (A, D, E, H, I) (Theme 1). For example in Study H, the rejection of foods mixed together on the plate was attributed to sensory sensitivity “parents described a picky eater as one who would not eat foods that are mixed…parents thought…the food combination may generate a sensory overload” (H), and in Study E this characteristic of fussy eating was attributed to personality traits (“*obsessive, compulsive sort of personality are the ones like, it has to be arranged on the plate like this and it can’t touch*”) (E).

#### Beliefs about hunger regulation

Finally, regarding hunger regulation, the belief that “*you can’t let them starve*” (B) and “eating something is better than nothing” (G) was highlighted by some study authors (B, D, G) and evident in participant quotes (A, I, J). In contrast to this, other parents were not concerned about letting their children get hungry and believed “the child would eat, if and when they became hungry enough” (I) (“*And if they don’t eat it, that’s fine…he’ll be hungry, not me*”) (I).

#### Feeding beliefs and feeding practices

These beliefs regarding self-efficacy, attributions and hunger regulation were associated with the use of different feeding practices. For example, authors highlighted the role of self-efficacy in implementing certain practices (such as not purchasing undesirable foods) (C, E, F and this relationship was evident in participant quotes in studies B, I and J). Parent attributions may relate to their feeding practices (A, E, F) although there was a lack of rich data to support this relationship and a reliance on second and third order interpretations, resulting in lower confidence in this finding. For example, parents may modify or disguise foods if they believe their fussy eater is sensitive to certain sensory properties of foods such as the taste, texture or colour of foods (A, B, F) “*I sneak green beans in the meatballs, and he’ll ask for a second helping”* (B)). Practices such as repeated exposure to disliked foods and role-modelling may be more likely if parents attribute fussy eating to modifiable environmental influences such as parent socialisation (e.g. “*if there is something she doesn’t like, I have to offer it again over the following weeks until she eats it*” (A). Finally, GRADE-CERQual indicated high confidence that parents’ beliefs about hunger regulation relate to their feeding practices, as this finding was reported across multiple studies (A, B, D, G, I, J). For example, offering alternative meals was associated with the belief that it is better to eat something rather than nothing (“*We’ll get some KFC but we’ll have to go to McDonalds and get them nuggets! (laughs)…so I’d rather them eat something than nothing”* (G)), whereas if parents were not concerned about children getting hungry they may be more likely only cook one meal (“*my role is, if I cook dinner and you don’t like it, then you don’t eat. So if she doesn’t like it then she doesn’t eat anything”* (I)).

The associations between child preferences, parent beliefs, and parent feeding practices were particularly evident in Studies E and F (using the same data set), which compared the beliefs and practices of parents with children in healthy preference, unhealthy preference and neophobic groups. Parents of children with healthy preferences had higher self-efficacy, were more likely to report the role of parent socialisation in influencing children’s preferences (“*it’s got a lot more to do with the environment around them and what they see other people doing*” (E)), and were more likely to use effective practices (“*we eat together, we eat the same food*” (F)). On the other hand, parents of children with unhealthy and neophobic preferences were more likely to have low self-efficacy *(“I can’t control what he likes”*(E)), report child factors like sensory sensitivity and stubbornness as influences of children’s preferences (“*I think it’s the texture of the skin. She doesn’t like the feel of it.”* (E)), and were more likely to report using less effective practices (“*you bribe her in every way possible*” (F)).

### Theme 5: Parent awareness of food preference development and effective feeding practices: possible associations with beliefs, practices and emotions

Parent awareness of food neophobia, food preference development and effective feeding practices was identified as a key metaphor (or finding) in Study G. Authors of this study reported that “primary caregivers of young children are unaware of food neophobia and food preference development” (G). Authors implied that a lack of awareness of how food preferences develop may be related to parents’ belief that eating ‘something is better than nothing’, as well as their use of ineffective practices such as repeated exposure to non-core foods (G). However, as indicated in Fig. 2, there was low confidence in these findings as there was inadequate data to identify a clear relationship between these factors. These beliefs and practices may also be explained by other factors (e.g. health concerns, desire to avoid conflict). In addition, this lack of awareness of food preference development may only be applicable to certain participant groups, such as parents of very young children (G).

Although there was limited data to support the finding, Study A also reported a change in children’s eating behaviours as sudden and unexpected “*He used to eat everything and overnight he started to be difficult*” (A) which may indicate that parents are unaware that these changes are likely to occur. Lack of awareness that these behaviours are, in fact, typical may lead parents to experience high levels of concern and guilt, “*He’ll get vitamin deficiency”; “Each time I wonder what I did wrong”* (A). In contrast, other parents did refer to food preference development “*their food preferences are actually emerging*” (A) and the use of effective practices such as role modelling and repeated exposure (A, F, G). Studies, particularly including parents of older children, provided examples of parents learning effective practices through trial and error (F, H, I, J) (*“I’ve done it before, and found out that that wasn’t the best way so I don’t, don’t make separate meals anymore”* (J)). Comparing these findings across studies indicates that awareness of neophobia, food preference development and effective practices varies significantly between parents and may develop over time as parents become more experienced. However, this theme relies on 3rd order (reviewer) interpretations, and further exploration regarding the role of parent awareness of food preference development and effective practices in contributing to the family experience of fussy eating behaviours is warranted.

## Discussion

This study has reviewed and synthesised findings from ten recent qualitative studies on childhood fussy eating behaviours. Meta-ethnography was used [24], involving a secondary analysis of the data presented in these studies. A conceptual model (Fig. 2) was produced illustrating the relationships between child characteristics (including fussy eating), parent feeding beliefs, feeding practices, mealtime emotions and parent awareness of food preference development, that have been proposed in the current qualitative literature.

In Theme 1, perceptions of fussy eating behaviours across ten qualitative studies were synthesised. A strength of the meta-ethnography approach is to identify and highlight findings hidden amongst individual studies [24]. In addition to the limited intake and variety of food, we identified less commonly reported characteristics of fussy eating, such as frequent changes in preferences [33, 34]. It is often reported that fussy eating peaks in early childhood [5, 6]. However, our synthesis demonstrated that fussy eating behaviours were still perceived to be common across three studies of parents with older children [34–36], even in general samples not specifically targeting ‘fussy eaters’. This supports findings of some quantitative studies in which fussy eating persisted in later childhood [37].

As illustrated in Fig. 2, the qualitative literature depicts parent feeding practices as a central component of the family experience of fussy eating behaviour. Authors of studies in this review often used language implying an effect of parent feeding practices on children’s eating behaviours (e.g. ‘strategies used by parents to influence their children’s preferences’). However, our secondary analysis actually found stronger qualitative data (using specific examples and quotes) illustrating changes in parent feeding behaviour due to their child, rather than changes in children’s behaviours as a result of parent feeding practices (both in the short term and in the long term). Our findings highlight that parent feeding practices do not exist independently and do not have a unidirectional influence on fussy eating. Instead they are embedded in a complex system, developing over time in response to a child’s behaviours, mealtime emotions and parent beliefs. This supports the adoption of a relational approach to studying fussy eating, in which both the parent and child are considered to have agency in contributing to the feeding relationship [38]. Our findings also support findings from other studies that show that genetics and other child factors [4, 10] play a role and fussy eating is not simply a product of parenting practices.

Numerous qualitative and quantitative studies have reported that fussy eating is associated with a negative emotional climate at mealtimes and that it contributes to parent stress and frustration [6, 9, 39, 40]. Our synthesis of qualitative studies identified two distinct ways in which fussy eating may relate to a poor emotional climate (Theme 3). Firstly, parents reported negative emotions that directly related to their child’s behaviour (e.g. child pushing plate away might make the parent feel concerned or frustrated). Secondly, fussy eating contributes to a negative emotional climate at mealtimes via parent feeding practices (e.g. pressure to eat increases conflict, cooking more than one meal increases stress). This distinction may be useful to consider in interventions that focus on emotional support for feeding [9]. Offering strategies that address both parents’ internal emotional responses to food refusal (e.g. parent anxiety, frustration) as well as the general mealtime emotional climate (e.g. stress, chaos, family conflict) may be beneficial in information based interventions [9]. Our model also suggests that negative emotions may impact fussy eating, mainly via the effect of negative emotions on feeding practices that reinforce fussy eating behaviours. This supports findings from quantitative work that affective factors (such as maternal psychological distress) are associated with certain parent feeding practices (such as not offering new foods) [18]. It is possible that these emotions are also driven by parent beliefs, however findings presented in the reviewed qualitative studies did not illustrate a clear relationship between beliefs and emotions.

Our synthesis identified three types of parent beliefs evident in the qualitative literature on fussy eating: feeding self-efficacy, attributions of fussy eating, and beliefs about hunger regulation (Theme 4). Self-efficacy has been considered an important factor in feeding, specifically in obesity prevention and breastfeeding research [41–43], but less is known about self-efficacy in relation to managing fussy eating behaviour. Although metaphors relating to self-efficacy were identified across multiple studies in this review, only one study specifically aimed to investigate self-efficacy beliefs [33]. However, the relationships between fussy eating, self-efficacy beliefs and parent feeding practices identified in our model support findings from cross-sectional quantitative studies that have reported higher levels of parent self-efficacy to be associated with increased variety of fruit and vegetables, more effective feeding practices, and lower likelihood of perceiving their child to be a picky eater [18, 44, 45]. Although self-efficacy was the term used by the original study authors, self-efficacy usually refers to control over one’s own behaviour, rather than the ability to influence another’s behaviour and implies that a child’s food intake and preferences can be controlled. The term ‘relational efficacy’ that has been proposed in recent parent-child socialisation literature [46] may be a more appropriate term in the feeding context.

The GRADE-CERQual assessment [30] also indicated relatively low confidence in the relationship between parent attributions (or beliefs about causes of fussy eating) and feeding practices, due to inadequate data to identify a clear relationship. Research on attributions of fussy eating is relatively new. Although a Parent Attribution for Child Eating Scale has been developed in a hospital feeding clinic setting [47], there has not been any quantitative research investigating how parent attributions of typical fussy eating behaviours relate to feeding practices. Therefore, it would be beneficial for future research to investigate both self-efficacy beliefs and parent attributions further, specifically how these beliefs develop and how they relate to parent feeding practices. The GRADE-CERQual assessment indicated higher confidence in the relationship between parent beliefs about hunger regulation and parent feeding practices. This supports findings by Tan & Holub [17], and Satter’s Division of Responsibility model in which supporting the child to regulate their own hunger and food intake is associated with eating competence and wider food acceptance [13, 48].

Theme 5 presents a relatively novel and under-researched finding that parent awareness of food preference development relates to their beliefs, practices and emotions. Although there was a lack of rich data resulting in low confidence in this finding in the GRADE-CERQual assessment [30], our synthesis suggests that parents’ awareness of neophobia, food preference development and effective practices varies considerably, and that parents’ awareness may develop over time as they learn from experience. Knowledge has been associated with feeding practices in previous quantitative research [49]. However, interventions that have focused on increasing parent knowledge in relation to feeding, for example through information leaflets, have had mixed results [9]. It would be useful for further qualitative research to explore parents’ awareness and knowledge of food neophobia, fussy eating and effective feeding practices, sources of parent knowledge (e.g. their own upbringing, experience of parenting, observations of other children/families, health professionals), and the contexts in which information-based interventions may be beneficial.

Together, these five themes form a line-of-argument synthesis, represented by the conceptual model in Fig. 2. This model illustrates the complex nature of the family experience of fussy eating behaviours. Our model supports some of the findings identified in Lafraire’s [7] model of factors that modulate food neophobia and picky/fussy eating as well as Koh’s [18] conceptual model of variety in fruit and vegetable intake. However, our model includes some additional factors specific to fussy eating (e.g. parent attributions of fussy eating). While previous models have focused on identifying predictors of food intake and eating behaviour [7, 18], the model presented in this review captures the components that determine how fussy eating behaviours are experienced by a family, specifically how fussy eating manifests (child characteristics), how it is perceived (parent beliefs and awareness), how it is experienced (mealtime emotions), and how it is managed (parent feeding practices). Fussy eating is not always disruptive to family meals [34] and even relatively severe fussy eating behaviours may not be problematic for a family depending on how they are perceived and managed.

The lack of a consistent and operational definition of fussy eating is one of the major limitations of research in this area, including the studies in this review [2, 34, 35, 50]. By synthesising parent perceptions and experiences of fussy eating across ten studies, we propose that fussy eating is an umbrella term describing the rejection of one or more food items, the limited intake or variety of foods, and/or frequent changes in food preferences due to novelty, sensory sensitivity, context/presentation of food, temperament/personality, age/developmental stage, and/or genetic and learned food preferences. Fussy eating can be expressed verbally or non-verbally (e.g. gestures, gagging, avoidance) and can (but does not always) have a perceived impact on the physical or psychological wellbeing of the child, parent or family. This definition may be useful for researchers, as current definitions often do not encompass the wide range of behaviours that ‘fussy eating’ can refer to, and do not clearly differentiate typical fussy eating behaviours from other forms of food refusal (e.g. due to allergy, medical conditions, religious or philosophical choices).

### Limitations of the qualitative literature on fussy eating

Our review of the qualitative literature found that most studies were conducted in the US and Australia and focused on toddlers and pre-schoolers, reflecting the belief that fussy eating peaks in early childhood [5]. Fathers’ perspectives were significantly underrepresented. We had hoped to include studies reporting the child perspective of fussy eating. Although some studies have qualitatively explored food choice with children [51], we did not identify any studies with children that focused on fussy eating or neophobia sufficiently to meet our inclusion criteria. The quality of studies (assessed using JBI Critical Appraisal Checklist [28]) included in this review was moderate to high. However, most studies failed to report philosophical perspectives or to provide a statement locating the researcher culturally or theoretically which makes it difficult to determine the impact that authors’ assumptions, knowledge and experiences may have on the research findings. Some factors that relate to fussy eating remain under-researched (e.g. parent awareness of food preference development and effective feeding practices, and attributions of fussy eating) and other factors that are known to be relevant to family mealtimes (such as parent feeding goals [52, 53]), were not evident in the qualitative studies eligible for inclusion in this review.

### Strengths and limitations of the qualitative synthesis

The meta-ethnography method was useful for identifying general patterns across studies and for highlighting findings hidden amongst individual studies that may have more meaning when related to the findings of other studies [24], specifically relationships between constructs. Rather than simply summarising existing knowledge, the meta-ethnography approach allowed us to build a new understanding of fussy eating (Fig. 2), based on the findings of individual studies whilst maintaining a focus on contextual factors such as study location, samples, and target-age range.

However, there are some limitations of this synthesis. Our literature search was restricted to English language publications. Some terms were not included in the database search (e.g. carer, caregiving, mum, dad) which may have resulted in identifying additional studies, although it is likely that any additional studies would have been identified during supplementary searches. Due to the diverse use of terminology and reporting in qualitative research [26, 27], there were some challenges in selecting studies for inclusion. We decided to only include studies with a primary aim or objective relating to fussy/picky eating or food neophobia. This may have resulted in relevant findings from other studies (e.g. on portion size, out of home eating etc.) being omitted from this review. In addition, the meta-ethnography approach is still evolving, resulting in differing interpretations of the steps involved and varied uses of meta-ethnography terminology [21]. We have reported the steps we carried out at each stage of the meta-ethnography process as transparently as possible (Table 1), have attempted to use the terminology as originally used by Noblit and Hare [24] and have followed both methodological and reporting guidelines provided by France and colleagues [31, 54] as closely as possible. Our findings represent the current literature in this area and are influenced by the methods, interview guides, interpretations and interests of the original study authors. Therefore, the transferability of these findings to contexts beyond those of the original studies is limited. Finally, it is not possible to infer cause and effect in cross-sectional qualitative research, but the relationships identified in this review are useful for developing hypotheses for future research.

### Recommendations for research and practice

It is recommended that future qualitative research on fussy eating focuses on the perspectives of both children and fathers, targets fussy eating behaviours in later childhood and adolescence, captures experiences of fussy eating across more diverse contexts, and improves the reporting standards of qualitative research methods [20]. As well as continuing research into parent feeding practices, it would be beneficial for attention to be focused on the more tentative components in the model (e.g. how parent awareness and attributions of fussy eating relate to parent feeding practices). The model can also be used to build hypotheses for longitudinal quantitative research to investigate, for instance, how feeding self-efficacy beliefs develop in the context of fussy eating and how parent feeding beliefs relate to parent feeding practices.

Targeting factors such as parent beliefs and mealtime emotions alongside parent feeding practices may improve the effectiveness of interventions aiming to prevent or resolve fussy eating related challenges [8, 9]. Our model (Fig. 2) may be of use to health professionals working in the area of fussy eating, in order to conceptualise how fussy eating is experienced by families, and the different types of beliefs and emotions that may need to be addressed with families to overcome fussy eating challenges.

## Conclusions

This review has used a meta-ethnography approach [24] to synthesise ten recently published qualitative studies on family perceptions, experiences and practices regarding fussy eating behaviours in typically developing children (aged one to young adult). Based on parent perceptions across ten studies, we propose that fussy eating is an umbrella term describing the rejection of one or more food items, the limited intake or variety of foods, and/or frequent changes in food preferences due to novelty, sensory sensitivity, context/presentation of food, temperament/personality, age/developmental stage, and/or genetic and learned food preferences. A conceptual model was produced, illustrating relationships between child characteristics (including fussy eating behaviours), parent feeding beliefs, parent feeding practices, mealtime emotions and parent awareness of food preference development, neophobia and effective feeding practices (Fig. 2). We found that child characteristics and parent feeding practices related to each other, supporting a relational approach to studying fussy eating in which both parents and children are considered to have agency in contributing to the feeding relationship [38]. Two distinct ways in which fussy eating relates to mealtime emotions were identified (directly and via feeding practices). Three distinct categories of parent beliefs were found to relate to parent feeding practices in the context of fussy eating (self-efficacy, attributions, and beliefs about hunger regulation). This review highlights areas for future qualitative research. Our model can be used to develop hypotheses for longitudinal quantitative studies and may be useful for health practitioners working with families experiencing fussy eating challenges.

## Data Availability

The datasets used and/or analysed during the current study are available from the corresponding author on reasonable request.
